# Voxel‐wise supervised analysis of tumors with multimodal engineered features to highlight interpretable biological patterns

**DOI:** 10.1002/mp.15603

**Published:** 2022-04-21

**Authors:** Thibault Escobar, Sébastien Vauclin, Fanny Orlhac, Christophe Nioche, Pascal Pineau, Laurence Champion, Hervé Brisse, Irène Buvat

**Affiliations:** ^1^ Laboratoire d'Imagerie Translationnelle en Oncologie (LITO) Institut Curie, Inserm, Université Paris‐Saclay Orsay France; ^2^ DOSIsoft SA Cachan France; ^3^ Department of Nuclear Medicine and Endocrine Oncology Institut Curie Saint‐Cloud France; ^4^ Department of Medical Imaging Institut Curie Paris France

**Keywords:** interpretability, machine learning, radiomics, sub‐region, voxel‐wise

## Abstract

**Background:**

Translation of predictive and prognostic image‐based learning models to clinical applications is challenging due in part to their lack of interpretability. Some deep‐learning‐based methods provide information about the regions driving the model output. Yet, due to the high‐level abstraction of deep features, these methods do not completely solve the interpretation challenge. In addition, low sample size cohorts can lead to instabilities and suboptimal convergence for models involving a large number of parameters such as convolutional neural networks.

**Purpose:**

Here, we propose a method for designing radiomic models that combines the interpretability of handcrafted radiomics with a sub‐regional analysis.

**Materials and methods:**

Our approach relies on voxel‐wise engineered radiomic features with average global aggregation and logistic regression. The method is illustrated using a small dataset of 51 soft tissue sarcoma (STS) patients where the task is to predict the risk of lung metastasis occurrence during the follow‐up period.

**Results:**

Using positron emission tomography/computed tomography and two magnetic resonance imaging sequences separately to build two radiomic models, we show that our approach produces quantitative maps that highlight the signal that contributes to the decision within the tumor region of interest. In our STS example, the analysis of these maps identified two biological patterns that are consistent with STS grading systems and knowledge: necrosis development and glucose metabolism of the tumor.

**Conclusions:**

We demonstrate how that method makes it possible to spatially and quantitatively interpret radiomic models amenable to sub‐regions identification and biological interpretation for patient stratification.

## 1 INTRODUCTION

Radiomics has been introduced in the 2010s to enhance the quantitative exploitation of medical images^1^ and corresponds to the extraction of a large number of mathematical descriptors from the image. These characteristics called “radiomic features” can be extracted through well‐defined mathematical expressions, so‐called “engineered features.” They can also be calculated by successive convolutional layers in deep convolutional neural networks, referred to as “deep features.”^2^, ^3^ By using these features as an input to machine learning and deep learning methods, classification and prediction models are designed, for instance to predict the response to a treatment.

Although supervised machine learning models have shown great potential in medical imaging, their clinical translation remains challenging.^4^ One reason is the difficulty in interpreting the models and understanding the information used to produce the output. Understanding models implies relating their output to some biological rationales and even possibly formulate new biological or medical hypotheses.

Whereas engineered features are mathematically well defined, the interpretation of models based on such features often remains challenging due to the complex definition of some features and to their weighted combination when building multivariate models. Moreover, in most studies, engineered features are directly calculated from a whole region of interest (ROI) with overall measurements, making it difficult to relate the outputs to a characterization at the voxel or sub‐region level. Mapping the contributions of the voxels to the model output would highlight the location of sub‐regions that are important for making a decision or a prediction and thus could help to increase transparency and interpretability. Several methods for such mapping have been proposed in the context of deep learning, for example.^5^, ^6^, ^7^, ^8^, ^9^, ^10^, ^11^ Yet, while these methods are promising, they have limitations, such as coarse resolution or sparse representation. More importantly, the complexity of the explained models and the high‐level abstraction of deep features limit their transparency. Indeed, knowing where the relevant information is in the image is helpful, but does not tell how this information is used.^12^ Finally, since complex models are often not inherently interpretable, explanations may not be faithful to what the original model computes.^12^, ^13^, ^14^ This could lead to potentially misleading explanations of what the model is actually based on. Therefore, methods that combine the spatial and the quantitative information related to the model outputs in a straightforward and reliable way are still needed.

Sub‐regional characterization using engineered voxel‐based radiomics could be useful in this context. Few reports describe such methods facilitating model interpretation. Wu et al.,^15^, ^16^ Xu et al.,^17^ and Even et al.^18^ used unsupervised clustering methods to identify tumor sub‐regions and associate them to patient outcome. Beaumont et al.^19^ used a random forest approach to predict local recurrence from baseline images thanks to locally calculated features and voxel‐wise ground truths. Vuong et al.^20^ investigated patch‐based radiomics with binary activation for tracing the spatial location of regions responsible for a given classification. To the best of our knowledge, although engineered radiomics is largely used especially when datasets are not amenable to deep radiomics, no approach has been proposed to quantitatively map, at the voxel level, the output of a model based on engineered radiomic features.

In this study, we propose an original mapping method of the outputs of a logistic model based on engineered features to enable its local and biological interpretation. In Section 2, we present the theory (Section 2.1), the dataset used in our experiments, and the experiments performed to test our method in the context of predicting the risk of lung metastases in soft tissue sarcomas (STS) based on positron emission tomography (PET)/computed tomography (CT) and magnetic resonance imaging (MRI) images (Section 2.2). The results are then described and discussed in Section 3 and Section 4.

## 2 MATERIALS AND METHODS

### 2.1 Theoretical background

This section describes how logistic regression can be used to bridge a probabilistic binary classification to a quantitative and interpretable voxel‐wise characterization map.

To allow for a mapping of the model output when using engineered radiomics, the features are initially extracted at the voxel level. A three‐dimensional (3D) cubic sliding window is used to compute the voxel‐wise features. For each position of the cube centered on voxel *v* inside the ROI, the radiomic features are calculated in this cube and the resulting values are assigned to *v* in the resulting feature maps.

The value of the *p*th feature assigned to voxel *v* is denoted $x_{p}^{\left(i,v\right)}$, which is a component of the voxel‐wise feature set $X^{\left(i,v\right)}$ for patient *i*. The *p*th radiomic feature $g_{p}^{\left(i\right)}$ for that patient *i* is obtained by averaging $x_{p}^{\left(i,v\right)}$ across all *v* inside the ROI.

(1) $$g_{p}^{\left(i\right)}=\frac{1}{Nv^{\left(i\right)}}\sum_{v=1}^{Nv^{\left(i\right)}}x_{p}^{\left(i,v\right)}$$

where $Nv^{\left(i\right)}$ is the total number of voxels within the tumor ROI of patient *i*.

Each tumor is thus described with a feature vector $G^{\left(i\right)}$ composed of *Np* features.

Using logistic regression, the probability $P^{\left(i\right)}$ for a given patient *i* to belong to class 1 is modeled as

(2) $$P^{\left(i\right)}=\frac{1}{1+e^{-D\left(G^{\left(i\right)}\right)}}$$

In this equation, *D* represents the linear decision function of *P*, with β_0_ its learned intercept and β the vector of learned coefficients associated to *G*, and is defined as

(3) $$\begin{array}{cc} D\left(G^{\left(i\right)}\right) & =\beta^{T}G^{\left(i\right)}+\beta_{0}\\ D\left(G^{\left(i\right)}\right) & =\sum_{p=1}^{\mathrm{Np}}\left(\beta_{p}g_{p}^{\left(i\right)}\right)+\beta_{0} \end{array}$$

where T represents the transpose operator.

The backprojection of β_0_ and β to $X^{\left(i,v\right)}$ at the voxel level yields a quantitative radiomic decision map (RDM) $DV^{\left(i\right)}$, mapping the individual participation $\mathrm{DV}(X^{\left(i,v\right)})\;$ of each and every voxel *v* to the probability for patient *i* to belong to class 1.

(4) $$\begin{array}{cc} \mathrm{DV}\left(X^{\left(i,v\right)}\right) & =\beta^{T}X^{\left(i,v\right)}+\beta_{0}\\ \mathrm{DV}\left(X^{\left(i,v\right)}\right) & =\sum_{p=1}^{\mathrm{Np}}\left(\beta_{p}x_{p}^{\left(i,v\right)}\right)+\beta_{0} \end{array}$$

The backprojection of β_0_ and β to $X^{\left(i,v\right)}$ preserves the probabilistic quantification. Indeed, due to the linear nature of the mean and of *D*, the mean value $\bar{DV^{\left(i\right)}}$ of $DV^{\left(i\right)}$ across all voxels in the ROI is equal to $D(G^{\left(i\right)})$.

(5) $$\begin{array}{cc} \bar{DV^{\left(i\right)}} & =\frac{1}{Nv^{\left(i\right)}\;}\sum_{v=1}^{Nv^{\left(i\right)}}\mathrm{DV}\left(X^{\left(i,v\right)}\right)\\ \bar{DV^{\left(i\right)}} & =\frac{1}{Nv^{\left(i\right)}}\sum_{v\;=\;1}^{Nv^{\left(i\right)}\;}\left(\beta^{T}X^{\left(i,v\right)}\right)+\beta_{0}\\ \bar{DV^{\left(i\right)}} & =\sum_{p=1}^{\mathrm{Np}}\left(\beta_{p}g_{p}^{\left(i\right)}\right)+\beta_{0}\\ \bar{DV^{\left(i\right)}} & =D\left(G^{\left(i\right)}\right)\\ \bar{\mathrm{DV}} & =\;D \end{array}$$

A more detailed development is reported in Equation (S1).

The decision function *D*, expressed as a function of the averaged local features *G*, is equal to the average of the local decision function, expressed as a function of the local features *X* across all voxels within the ROI for each patient. As such, we can express the probability for a given patient *i* to belong to class 1 directly at the voxel level using

(6) $$P^{\left(i\right)}=\frac{1}{1+e^{-\left(\frac{1}{Nv^{\left(i\right)}}\sum_{v=1}^{Nv^{\left(i\right)}}\left(\beta^{T}X^{\left(i,v\right)}\right)+\beta_{0}\right)}}$$

The proposed method thus produces RDMs that quantify the contribution of each voxel to the patient classification, highlighting the most contributory sub‐regions within the ROI.

Some features, such as the shape features, do not have any voxel‐level counterpart, while they can still contribute to a decision function that then reads as

(7) $$\begin{array}{ccc} D\left(\left[G^{\left(i\right)};G{'}^{\left(i\right)}\right]\right) & = & \beta^{T}G^{\left(i\right)}+\beta {'}^{T}G{'}^{\left(i\right)}+\beta_{0}\\ D\left(\left[G^{\left(i\right)};G{'}^{\left(i\right)}\right]\right) & = & \frac{1}{Nv^{\left(i\right)}}\sum_{v=1}^{Nv^{\left(i\right)}}\left(\beta^{T}X^{\left(i,v\right)}\right)+\beta {'}^{T}G{'}^{\left(i\right)}+\beta_{0} \end{array}$$

where $G{'}^{\left(i\right)}$represents the vector composed of Np'features $g{'}_{p'}^{\left(i\right)}$without any local counterpart that is concatenated to $G^{\left(i\right)}$, and $\beta {'}_{p'}$the learned coefficients associated with these features.

Similar to Equation (6), the probability for a given patient *i* to belong to class 1 can be expressed as

(8) $$P^{\left(i\right)}=\frac{1}{1+e^{-\left(\frac{1}{Nv^{\left(i\right)}}\sum_{v=1}^{Nv^{\left(i\right)}\;}\left(\beta^{T}X^{\left(i,v\right)}\right)+\beta {'}^{T}G{'}^{\left(i\right)}+\beta_{0}\right)}}$$

When the model includes features without voxel‐level counterpart, only part of the model is explained by the RDMs. The coefficients associated with features that can be mapped indicate the importance of these features compared to the ones that cannot be mapped.

### 2.2 Experiments

#### 2.2.1 Patients and data

We used a publicly available dataset of 51 STS patients for whom fluorodeoxyglucose (^18^F) ([^18^F]‐FDG) PET, CT, T1, and fat‐suppressed T2 MRI images, ROIs, clinical, and follow‐up information were available.^21^ During the follow‐up period, 19 patients developed lung metastases and 32 did not. The task was to predict the occurrence of lung metastases at 2 years. PET/CT images were all acquired at the McGill University Health Centre using the same scanner (Discovery ST, GE Healthcare, Waukesha, WI, USA) for the 51 patients. MRI scans were acquired as part of routine care for each patient, with heterogeneous protocols across patients. T1‐weighted MRI images were available for all 51 patients. Two types of fat‐suppressed T2 sequences were acquired, namely fat‐saturated T2‐weighted (*n* = 26 patients) and short tau inversion recovery (*n* = 25). The provided tumor ROI had been manually drawn by an expert radiation oncologist on fat‐suppressed T2 images and propagated to PET, CT, and T1 images after rigid registration. Detailed information is provided by Vallières et al.^21^

#### 2.2.2 Image processing and feature extraction

##### MRI preprocessing

T1 images were corrected for bias field with the N4ITK algorithm,^22^ with default parameters and body mask as the region for bias field estimation. No correction was possible on fat‐suppressed T2 images because there was no significant signal outside the tumor ROI to estimate the bias field.

In MRI T1 and T2 images, a voxel value cannot be readily interpreted in terms of physical quantity, and the same tissue type can yield different voxel values between different acquisitions even when the images are acquired in the same patient using the same protocol settings. An adapted version of the White Stripe method^23^ was used to linearly scale the images based on the fat as a reference tissue for all T1 images. Spheres (average volume ± 1 standard deviation (SD) of 239 ± 52 mm^3^) were manually drawn in fat tissue for each patient (23 ± 9 spheres per patient). For each patient, every voxel was linearly transformed so that the mean value over all fat spheres was 0 with a SD of 1.

(9) $$Iw_{v}=\frac{I_{v}-\mu_{\mathrm{fat}}}{\sigma_{\mathrm{fat}}}$$

where $I_{v}$ is the intensity of each voxel *v* in the N4ITK corrected T1 image, σ_fat_ and μ_fat_ are the SD and the mean intensity within the reference fat tissue in the image, and $Iw_{v}$ is the normalized value at voxel *v*.

As no reference tissue could be used to normalize fat‐suppressed T2 images, a *z*‐score normalization was used based on each tumor ROI, so that the mean value in each tumor was 0 with a SD of 1 after normalization.

(10) $$Iz_{v}=\frac{I_{v}-\mu_{\mathrm{tum}}}{\sigma_{\mathrm{tum}}}$$

where $I_{v}$ is the intensity of each voxel *v* in the fat‐suppressed T2 image, σ_tum_ and μ_tum_ are the SD and the mean intensity within the tumor ROI, and $Iz_{v}$ is the *z*‐score normalized value at voxel *v*. The difference between normalization Equations (9) and (10) is that Equation (9) preserves inter‐patient variabilities of the signal intensity between tumors whereas Equation (10) does not.

##### Radiomic feature maps calculation

All images were resampled to isotropic voxels before feature extraction using third‐order B‐spline interpolation. PET images were expressed in standardized uptake value (SUV) units, resampled to 3 mm × 3 mm × 3 mm voxels and a fixed bin size discretization^24^ of 0.3125 SUV was used.^25^ CT images expressed in Hounsfield units (HU) were resampled to 1 mm × 1 mm × 1 mm voxels and a fixed bin size of 10 HU was used. Preprocessed T1 and fat‐suppressed T2 images were resampled to 1 mm × 1 mm × 1 mm voxels and bin sizes were set so that 128 bins were defined between the minimum and the maximum voxel value across the whole cohort which corresponded to 0.1668 for T1 images and 0.05611 for fat‐suppressed T2 images. CT voxels with values less than ‐230 HU or greater than 600 HU were excluded from the CT ROI to limit the presence of air and bone voxels in the ROI, while keeping values possibly associated with tumor hypodensities and calcifications.

First‐order, gray‐level co‐occurrence matrix, gray‐level dependence matrix, gray‐level run length matrix, and neighboring gray tone difference matrix radiomic features were locally extracted within all tumor ROI using a 3D sliding window of nine voxels in each dimension, leading to 308 radiomic feature maps per patient (77 feature maps per modality).

##### ROI‐feature calculation

As defined in Equations (1) and (2), the average value over the ROI was calculated for each feature to yield two ROI‐feature vectors of 154 components each per patient, one vector from the PET/CT feature maps (composed of 77 ROI‐features from the PET and 77 ROI‐features for the CT) and another from the MRI feature maps (composed of 77 ROI‐features from the T1 and 77 ROI‐features from the fat‐suppressed T2). In addition, volume and shape features were calculated from the CT resampled segmentation mask (1 mm × 1 mm × 1 mm voxels), producing 14 additional features that were added to the PET/CT and MRI features to yield two 168 features vectors.

All image features used in this work are listed in Table S1 and their definition can be found at https://pyradiomics.readthedocs.io/.^26^

#### 2.2.3 Machine learning probabilistic classification

In this section, PET/CT and MRI features were used separately. We created two separate models in order to test our method in two different clinically realistic settings. The main objective was to determine if the models were based on common areas, areas specific to the information carried by each modality, or a combination of both.

##### Multicollinearity reduction

Many radiomic features can be highly correlated hence collinear. Collinearity is a linear association between two features whereas multicollinearity refers to a situation in which more than two features are linearly related. Collinearity and multicollinearity can be seen as redundancies in the data and could adversely affect the stability of generalized linear models such as logistic regression. To cope with that problem, unsupervised feature selection was first performed using pairwise Pearson correlation on the PET/CT and the MRI feature sets. A maximum absolute Pearson *R* threshold was initialized to 1. As long as there was perfect multicollinearity in the data (null Pearson correlation matrix determinant), this threshold was iteratively decreased by a step of 0.001. During this process, if two features were correlated so that their absolute Pearson *R* value exceeded the threshold, the feature with the highest mean absolute *R* value with the other features was removed. Then, feature selection was performed by calculating the variance inflation factor (VIF),^27^ which estimates how much the variance of a regression coefficient increases due to the presence of multicollinearity. Let $G_{p}$ be the *p*th ROI‐feature vector composed of $g_{p}^{\left(i\right)}$ for all patients in the dataset. For each ROI‐feature $G_{p}$, we can compute $\mathrm{VI}F_{G_{p}}$ by linearly regressing it against the other features in the dataset.

(11) $$\mathrm{VI}F_{G_{p}}=\frac{1}{1-R_{G_{p}}^{2}}$$

where $R_{G_{p}}^{2}$ represents the coefficient of determination of the linear regression associated to $G_{p}$.

The VIF quantifies how much each feature introduces redundancy in the data while considering all the features together, unlike pairwise correlations that are based on two‐by‐two comparisons. Highly redundant features were thus removed by dropping the feature with the highest VIF iteratively until the maximum VIF was <10 in the feature set.^27^

##### Multivariate modeling

From this stage, the PET/CT and MRI models are, respectively, denoted as M1 and M2.

For both PET/CT and MRI, least absolute shrinkage and selection operator (LASSO) (also denoted L1) regularized logistic regression was used to model the probability of lung metastasis occurrence with the cost‐sensitive balanced cross‐entropy as a loss function to account for data imbalance. The features resulting from the multicollinearity reduction step were further selected through a forward sequential wrapper using a metric adapted from the stratified Brier scores.^28^ We defined the “average stratified Brier score” (ASB) measuring the calibrated and continuously defined accuracy of the modeled probability, with equal importance between class 1 (lung metastasis occurrence) and class 0 (no lung metastasis occurrence), as follows:

(12) $${\mathrm{SB}}_{C1}=\frac{1}{N_{C1}}\sum_{i=1}^{N_{C1}}{\left(y^{\left(i\right)}-P^{\left(i\right)}\right)}^{2}\left[\;y^{\left(i\right)}=\;1\right]$$

(13) $${\mathrm{SB}}_{C0}=\frac{1}{N_{C0}}\sum_{i=1}^{N_{C0}}{\left(y^{\left(i\right)}-P^{\left(i\right)}\right)}^{2}\left[\;y^{\left(i\right)}=\;0\right]$$

(14) $$\mathrm{ASB}=1-\frac{{\mathrm{SB}}_{C0}+{\mathrm{SB}}_{C1}}{2}$$

where *N*_C1_ and *N*_C0_ are the number of patients belonging to classes 1 and 0, respectively, in the data subset from which the score is computed, and SB_C1_ and SB_C0_ are the associated stratified Brier score where $y^{\left(i\right)}$ and $P^{\left(i\right)}$ are the outcome and the predicted probability of belonging to class 1 for patient *i*, respectively. […] is an Iverson bracket, which equals 1 when the condition within the brackets is true and 0 otherwise. The ASB score is defined for all patients from 0 to 1. A perfect model yields a mean ASB score of 1 whereas a model that always predicts the wrong class yields a mean ASB score of 0. A totally underfitted model which always predicts a probability of 0.5 yields a mean ASB score of 0.75, and a dummy model predicting the majority class yields a mean ASB score of 0.5.

A grid‐search approach was used to determine the optimal regularization term *C* of the LASSO and the number of forward selected features to be kept. *C* corresponds to the inverse of the regularization strength usually denoted as λ or α. Thus, a lower value of *C* means a higher regularization. Ten values were defined for *C* from 0.1 to 100 on a log_10_ scale. For each value of *C*, the forward selection procedure was performed using 200× 5‐fold repeated stratified cross‐validation with the reduced feature set based on the VIF. Training samples were used to scale the features using the *z*‐score normalization at each iteration of the cross‐validation procedure. The mean and SD of the ASB score were saved with the associated feature subset at each iteration of the forward selection procedure. The retained *C* parameter and feature subset were manually selected based on a tradeoff between the maximization of the mean ASB score and the minimization of its SD and coefficient of variation, while favoring the most parsimonious (few features) and regularized (low *C*) models.

To test whether our approach yielded over‐optimistic results by fitting data with noise, a permutation test was performed.^29^ The whole machine learning pipeline including the forward selection and the grid‐search optimization was repeated 200 times performing random permutations of the class labels at each iteration. For every iteration, the best grid‐search mean ASB score was saved, leading to a null distribution of the 200 best cross‐validated scores. This distribution shows the estimated performance when there is no real relationship between features and labels. Based on this null distribution, empirical *p*‐value associated with the ASB score observed for the models obtained for the correct labels could be calculated.

In addition to their mean ASB score, the standard Brier score loss, the mean receiver operating characteristic (ROC) curve, and its associated mean area under the curve (AUC) with SD were computed as figures of merit.

##### Bootstrap aggregation and comparison to usual biomarkers

Bootstrap aggregation, usually shortened as “bagging,” prevents overfitting by reducing the variance of the final classifier in comparison to a final classifier trained on the whole dataset. Therefore, 1000 bootstrap samples were drawn to build the models. The decisions functions’ coefficients of the 1000 bootstrap models were averaged to obtain the final linear decision functions of M1 and M2, denoted as *D*_M1_ and *D*_M2_.

The prediction performance of the two models was compared with that of usual biomarkers. During the bootstrap resampling of the bagging procedure, training samples were used at each iteration to scale the features using the *z*‐score normalization, and the ROC AUC were computed based on out‐of‐bag (OOB) samples for model predictions as well as for anatomical tumor volume (ATV), SUVmax, metabolic tumor volume (MTV), and total lesion glycolysis (TLG).

##### Decision maps and signature generation

The mean $\mu_{G_{p}}$and SD $\sigma_{G_{p}}$over all patients of each feature $G_{p}$involved in final models M1 and M2 were used to normalize the corresponding feature maps of each patient *i* in the dataset.

(15) $$z_{p}^{\left(i,v\right)}=\frac{x_{p}^{\left(i,v\right)}-\mu_{G_{p}}}{\sigma_{G_{p}}}$$

where $z_{p}^{\left(i,v\right)}$denotes the normalized value of the *p*th voxel‐wise feature computed at voxel *v* for patient *i*, and $x_{p}^{\left(i,v\right)}$its original value.

After resampling all feature maps on a common grid of 1 mm × 1 mm × 1 mm voxels using third‐order B‐spline interpolation, the RDMs ${\mathrm{DV}}_{M1}^{\left(i\right)}$, and ${\mathrm{DV}}_{M2}^{\left(i\right)}$were obtained for each patient iby backprojecting the ROI‐features’ coefficients at the voxel level.

#### 2.2.4 Practical implementation

All images and masks were saved in NIfTI‐1 format. Using Python (version 3.7.10), the bias field correction for MRI T1 images was performed with the N4ITK algorithm implementation of the SimpleITK library (version 2.0.2).^30^ MRI intensity normalization used spheres manually drawn in fat tissue with LIFEx software (version 6.31).^31^ Basic image operations and manipulations were performed using the Python libraries NumPy (version 1.16.6),^32^ NiBabel (version 2.5.1),^33^ and Nilearn (version 0.5.2).^34^ Radiomic features were computed using the Image Biomarker Standardization Initiative^24^ compliant Python library PyRadiomics (version 2.2.0).^26^ The multicollinearity reduction was performed using R (version 4.0.2) with the libraries Car (version 3.0‐9)^35^ and Caret (version 6.0‐90).^36^ The machine learning steps used the Python libraries Scikit‐Learn (version 0.20.4)^37^ and Pandas (version 0.25.3).^38^, ^39^ The logistic models were trained using the Liblinear^40^ deterministic coordinate descent algorithm, with LASSO (L1) penalty, a tolerance of 0.0001 as stopping criteria, and a maximum number of iteration equal to 100. We used LIFEx for all visualizations and interpretations.

All processing and analysis steps were run on Linux Ubuntu 20.04.2, on a Dell Precision Tower 7920 with 128 Gb of RAM memory, a 2 × 12‐Core Intel Xeon Silver 4214 64 bit, and a 16 Gb Nvidia Quadro RTX 5000 graphic card.

## 3 RESULTS

### 3.1 Feature extraction and multicollinearity reduction

**FIGURE 1:**
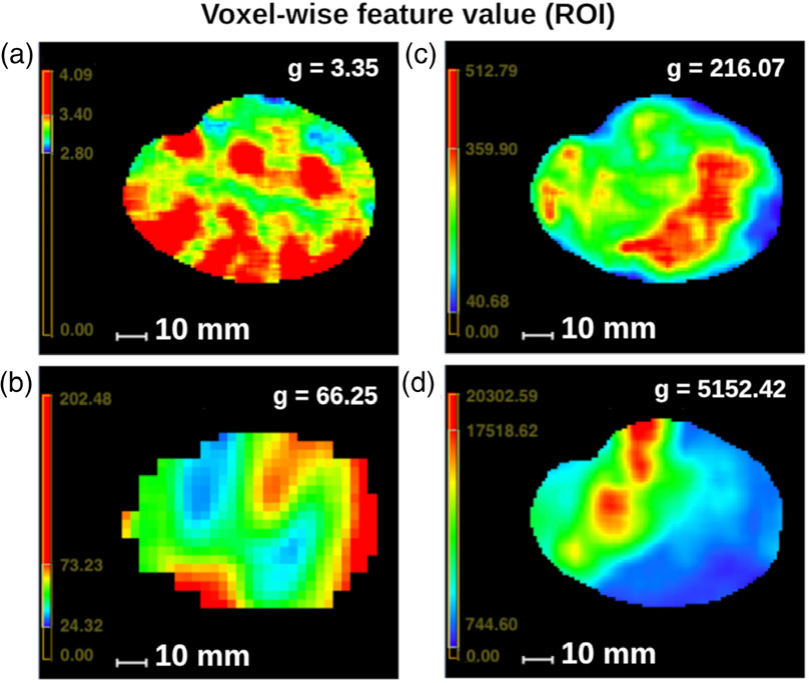
Example of engineered radiomic feature maps. As the models were trained by taking the mean values inside the region of interest as inputs, there was no need to resample all the feature maps on a common grid at this stage. The feature maps then had different spatial resolution (3 mm × 3mm × 3 mm for positron emission tomography (PET), 1 mm × 1 mm × 1 mm for computed tomography (CT) and magnetic resonance imaging (MRI)). (a) CT first‐order entropy, (b) PET gray‐level co‐occurrence matrix (GLCM) contrast, (c) T1 gray‐level dependence matrix (GLDM) gray‐level non‐uniformity (GLNU), and (d) fat‐suppressed T2 gray‐level run length matrix (GLRLM) long run high gray‐level emphasis (LRHGLE)

Four examples of voxel‐wise feature maps are shown in Figure 1 with their respective mean ROI‐feature value for a single patient, highlighting a variety of different patterns.

A total of 25 (21 averaged voxel‐level features and four shape features that cannot be mapped) and 26 (22 averaged voxel‐level features and four shape features) ROI‐features over 168 were selected from PET/CT and MRI, respectively, through the multicollinearity reduction step. The VIF value of the selected features is reported in Table S2. Figure S1 represents the Pearson correlation matrices of these features for PET/CT (a) and MRI (b). As expected, several features were found to be redundant at the ROI level, and only a fraction of them were retained after reducing the multicollinearity.

### 3.2 Multivariate modeling

**FIGURE 2:**
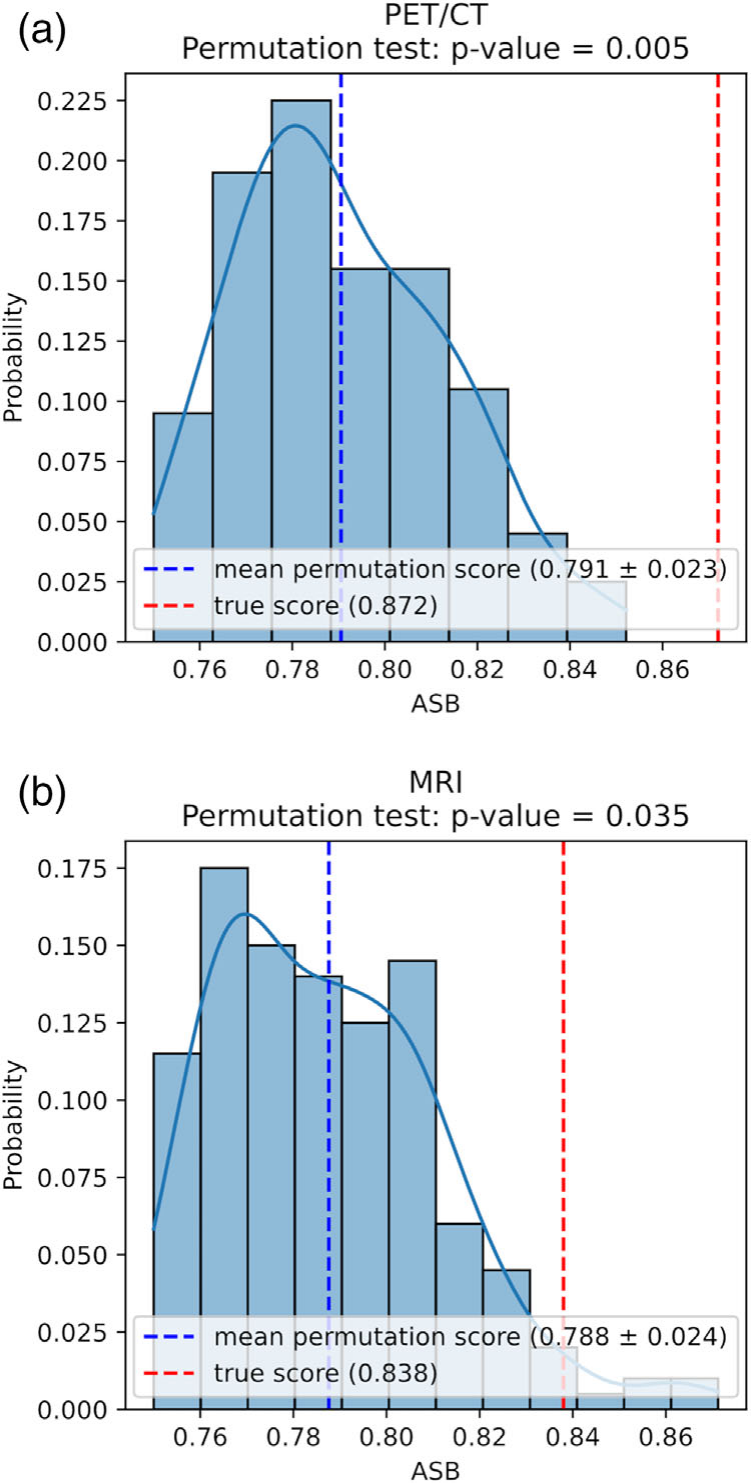
Average stratified Brier score permutation test distribution for M1 and M2 model building settings. (a) Positron emission tomography (PET)/computed tomography (CT) and (b) magnetic resonance imaging (MRI)

The null distributions of the 200 random models from the permutation tests are shown in Figure 2 together with the real cross‐validated performance of the M1 (a) and M2 (b) models. Five features were retained for PET/CT and MRI both with C=2.2 through the grid‐search procedure. The associated mean ASB scores (±1 SD) were 0.872 ± 0.056 (*p*‐value = 0.005) for PET/CT and 0.838 ± 0.065 (*p*‐value = 0.035) for MRI, significantly higher than those of the random models in both cases. Model building results are summarized in Table 1.

**TABLE 1:** Cross‐validated performance for the grid‐search forward selection and least absolute shrinkage and selection operator (LASSO) *C* parameter optimization

Model building settings	M1	M2
*C*	2.2	2.2
Number of selected features	5 (1 shape feature)	5 (1 shape feature)
ASB (±1 SD)	0.872 ± 0.056	0.838 ± 0.065
Brier score loss (±1 SD)	0.133 ± 0.057	0.167 ± 0.068
ROC AUC (±1 SD)	0.910 ± 0.094	0.853 ± 0.115

Abbreviations: ASB, average stratified Brier score; AUC, area under the curve; ROC, receiver operating characteristic; SD, standard deviation.

### 3.3 Final bagging models and comparison to usual biomarkers

The bagging linear decision functions$\;D_{M1}$ and *D*_M2_ are reported in Equations (16) and (17) with the SD associated to each feature across the 1000 bootstrap samples.

(16) $$\begin{array}{cc} D_{M1}=\; & -0.653\;\left(\pm 0.623\right)\times {\mathrm{CT}}_{\mathrm{GLD}M_{\mathrm{LDLGLE}}}\\ & +1.711\;\left(\pm 0.745\right)\times \mathrm{PE}T_{\mathrm{FIRST}\;\mathrm{ORDE}R_{\mathrm{MINIMUM}}}\\ & +2.655\;\left(\pm 0.907\right)\times \mathrm{PE}T_{\mathrm{FIRST}\;\mathrm{ORDE}R_{\mathrm{SKEWNESS}}}\\ & +1.469\;\left(\pm 0.600\right)\times \mathrm{PE}T_{\mathrm{GLC}M_{\mathrm{CORRELATION}}}\\ & +0.953\;\left(\pm 0.710\right)\times \mathrm{SHAP}E_{\mathrm{ELONGATION}}\\ & -0.673\;\left(\pm 0.428\right) \end{array}$$

(17) $$\begin{array}{ccc} D_{M2} & = & \;-1.325\;\left(\pm 0.735\right)\times T1_{\mathrm{FIRST}\;\mathrm{ORDE}R_{\mathrm{ENERGY}}}\\ & & -\;1.729\;\left(\pm 0.698\right)\times T1_{\mathrm{GLD}M_{\mathrm{SDLGLE}}}\\ & & +\;1.032\;\left(\pm 0.470\right)\times \mathrm{fat}-\mathrm{supressed}\\ & & -\;T2_{\mathrm{FIRST}\;\mathrm{ORDE}R_{\mathrm{RMS}}}+1.895\;\left(\pm 0.731\right)\\ & & \times \;\mathrm{fat}-\mathrm{supressed}-T2_{\mathrm{FIRST}\;\mathrm{ORDE}R_{\mathrm{ENERGY}}}\\ & & +\;1.197\;\left(\pm 0.577\right)\times \mathrm{SHAP}E_{\mathrm{SPHERICITY}}\\ & & -\;0.857\;\left(\pm 0.444\right) \end{array}$$

**FIGURE 3:**
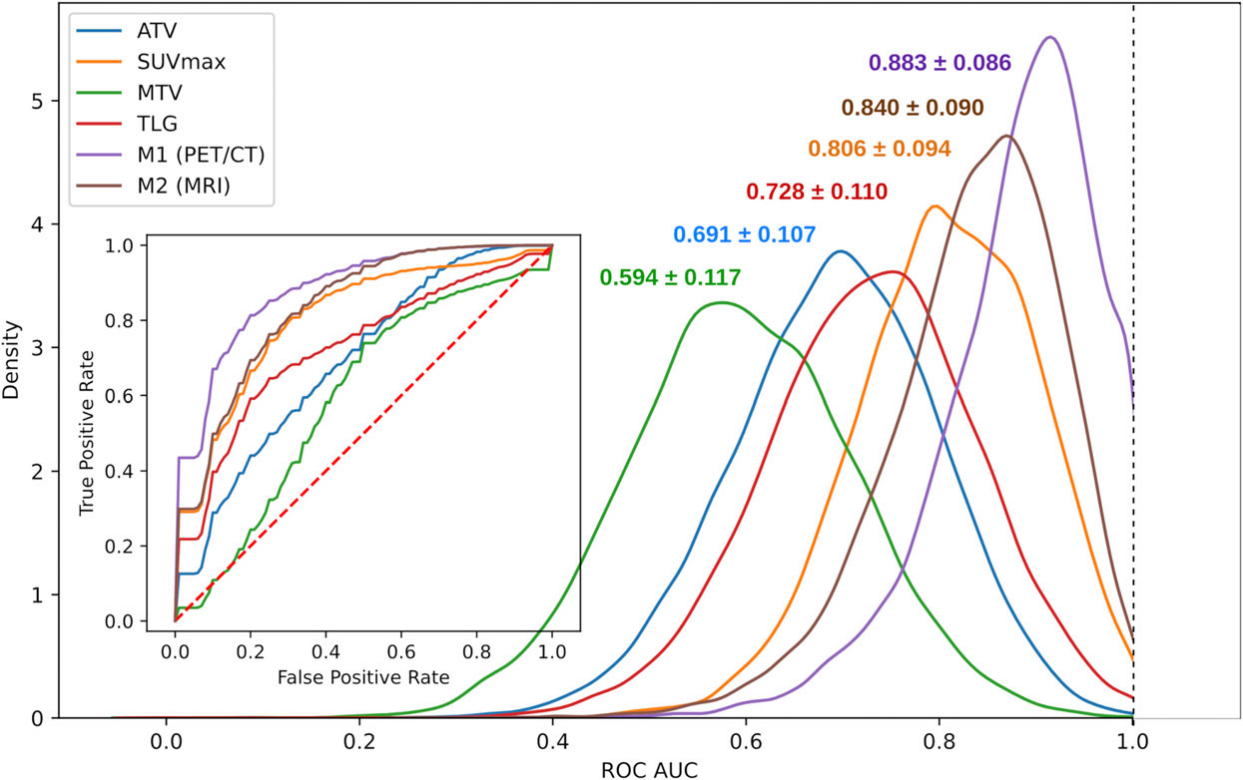
Probability density function of the out‐of‐bag (OOB) receiver operating characteristic (ROC) area under the curve (AUC) for M1 (positron emission tomography (PET)/computed tomography (CT)), M2 (magnetic resonance imaging (MRI)), anatomical tumor volume (ATV), SUVmax, metabolic tumor volume (MTV), and total lesion glycolysis (TLG). The average ROC curves associated with these distributions are reported in the left sub‐figure

Figure 3 shows the probability density function of the OOB ROC AUC distributions for M1 and M2 predictions, SUVmax, TLG, ATV, and MTV. Models and biomarkers bootstrap OOB ROC AUC are summarized in Table 2.

**TABLE 2:** Bootstrap out‐of‐bag (OOB) receiver operating characteristic (ROC) area under the curve (AUC) for M1 (positron emission tomography (PET)/computed tomography (CT)), M2 (magnetic resonance imaging (MRI)), anatomical tumor volume (ATV), SUVmax, metabolic tumor volume (MTV), and total lesion glycolysis (TLG)

OOB ROC AUC	M1	M2	ATV
Mean (±1 SD)	0.883 ± 0.086	0.840 ± 0.090	0.691 ± 0.107
95% CI	[0.660, 1.000]	[0.622, 0.974]	[0.472, 0.890]
Maximum PDF (mode)	0.908	0.858	0.703

OOB ROC AUC	SUVmax	MTV	TLG
Mean (±1 SD)	0.806 ± 0.094	0.594 ± 0.117	0.728 ± 0.110
95% CI	[0.612, 0.971]	[0.361, 0.818]	[0.501, 0.931]
Maximum PDF (mode)	0.789	0.569	0.749

Abbreviations: CI, confidence interval; PDF, probability density function; SD, standard deviation.

### 3.4 Decision maps

**FIGURE 4:**
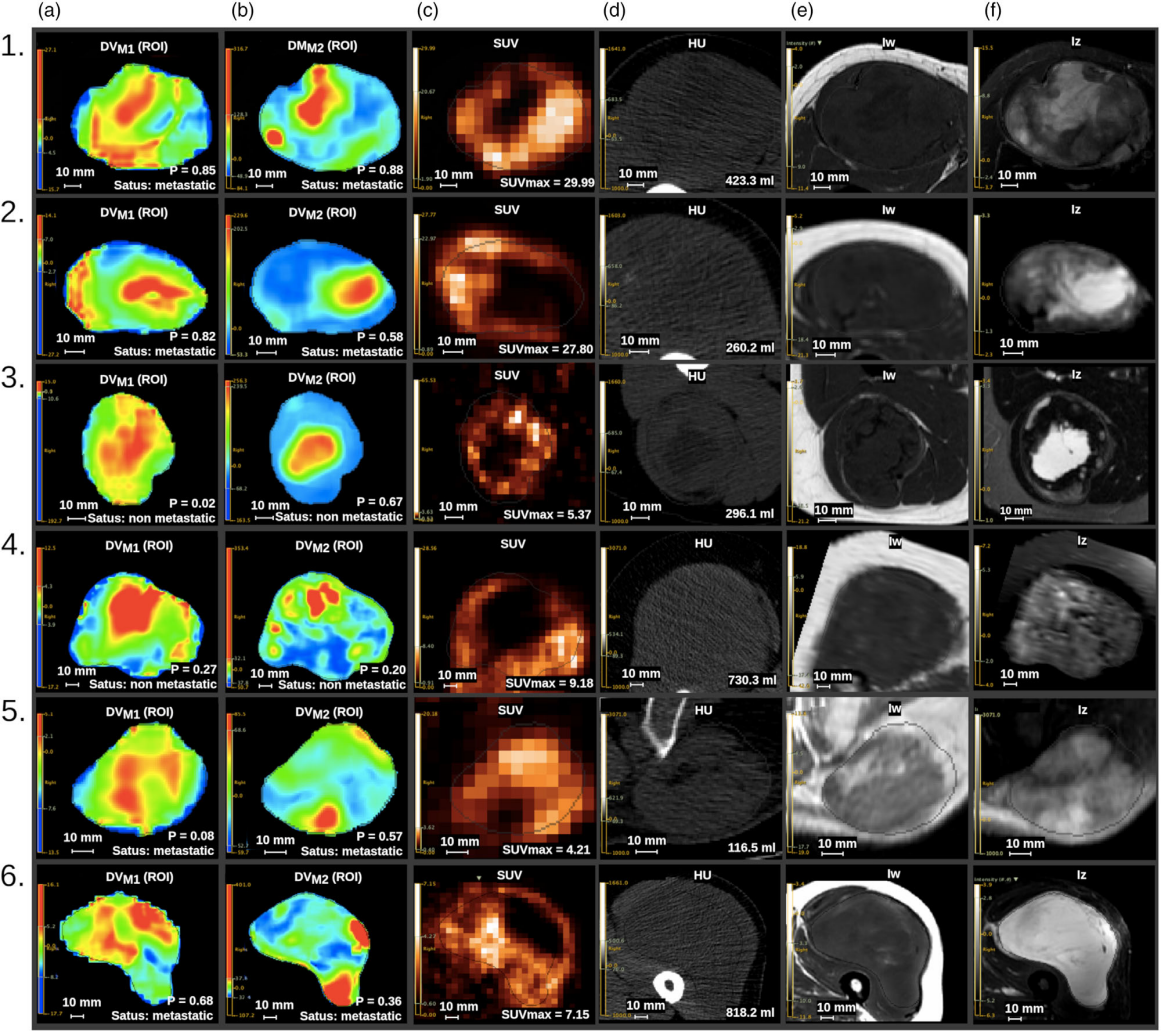
Slice examples of radiomic decision maps *D*V_M1_ (a) and *D*V_M2_ (b), positron emission tomography (PET) (c), computed tomography (CT) (d), T1 (e), and fat‐suppressed T2 (f) images for six patients

Representative slices examples of RDMs $DV_{M1}$(a) and $DV_{M2}$(b), PET (c), CT (d), T1 (e), and fat‐suppressed T2 (f) images are shown in Figure 4 for six patients (1–6). The RDMs $DV_{M1}$and ${\mathrm{DV}}_{M2}$revealed predictive patterns that are interpretable and consistent across patients. In particular and supported by Equations (16) and (17), $DV_{M1}$highlighted high FDG uptake sub‐regions, substantial and homogeneous tumor regions with low metabolism, and some hypodense sub‐regions. The sub‐regions highlighted by the $DV_{M2}$maps showed overall good colocalization with the ones that were hypodense and non‐FDG‐avid in the $DV_{M1}$maps. This corresponds to low‐signal sub‐regions in T1 and high‐signal sub‐regions in fat‐suppressed T2 images. Biologically, these sub‐regions correspond to suspected necrosis. In $DV_{M1}$maps, the sub‐regions characterized by focal and heterogeneous high FDG uptake most of the time included the SUVmax voxel.

Patient 1 risk was well predicted with a high probability from both M1 (0.85) and M2 (0.88), consistent with the high SUVmax (29.99) and the large necrotic sub‐region seen in the tumor. With a well‐predicted risk for both M1 (0.82) and M2 (0.58), patient 2 PET image showed a smaller necrotic sub‐region but still a high SUVmax (27.80), consistent with the lower predicted probability for M2 than for M1. Necrotic volume of patients 2 and 3 were comparable, with a lower SUVmax (5.37) for patient 3. This could explain the lower predicted probability for M1 (0.02) than for M2 (0.67), yielding a false positive for M2. M1 (0.27) and M2 (0.20) well predicted similar low probability for patient 4. The predicted probability for patient 5 led to a false negative for M1 (0.08) and a true positive for M2 (0.57), still consistent with the relatively low SUVmax (4.21) for this patient. Last, the predicted probabilities for patient 6 with a SUVmax of 7.15 and a large necrotic volume led to a true positive for M1 (0.67), illustrating its superiority over SUVmax in this case, despite their consistency.

These findings suggest that two biological local image patterns were associated with the risk of lung metastasis occurrence in this dataset: the development of necrosis in the tumor and its high glucose metabolism.

### 3.5 Surrogate model

From our reading of the RDMs and the model equations, and to assess the validity of our interpretations, we built a simplified surrogate model from M1, namely M1ʹ. We engineered simpler and more easily interpretable features with the aim of describing the necrotic development inside the anatomical volume of the tumor with PET/CT images. We computed the absolute volume (*V*) and the relative volume (*rV*) over ATV that were characterized either by a low metabolism (<40% SUVmax in PET), a hypodense signal (<20 or <30 HU in CT), or a combined measure of these two patterns using the union or the intersection operators. The log_10_ transformation was also applied to these new features as well as to ATV, SUVmax, MTV, TLG, and the shape features to increase the size of the feature set, account for skewed distributions, and allow for more flexibility for the modeling. We finally built M1ʹ by training a logistic model with the predicted output of M1 as a target, following the same machine learning procedure but using only these features. All the new features are listed in Table S3 with their definition.

**FIGURE 5:**
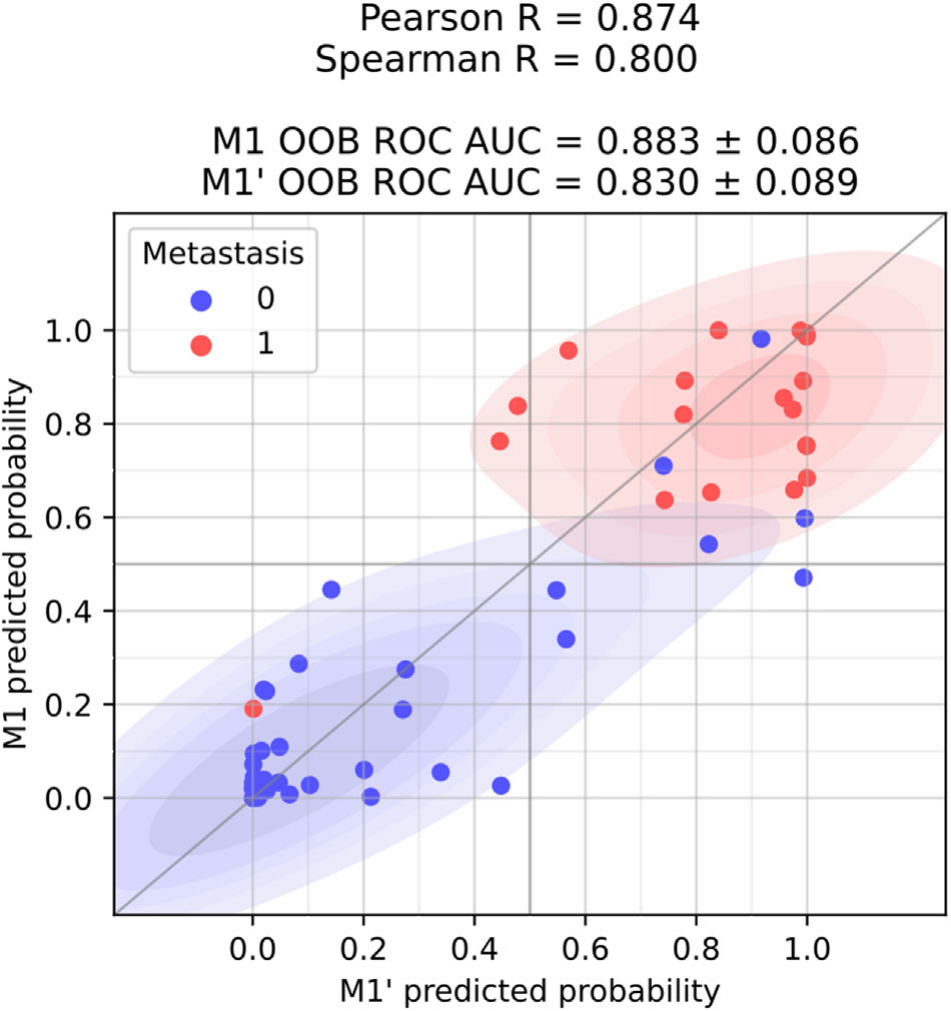
Joint scatter and kernel density estimation plot comparing M1ʹ and M1 probabilistic outputs on the whole dataset. The color of the dots represents the true label of the corresponding patients (blue: no lung metastasis occurrence, red: lung metastasis occurrence)

As a result, three features were automatically selected to approximate the M1 predictions: log_10_(SUVmax), log_10_(HYPODENSE_20 HU_ ∪ INACTIVE_FDG_ *V*), and SHAPE_ELONGATION_. The bagging linear decision function of M1ʹ,$\;D_{M1^{\prime }}$, is reported in Equation (18) with the SD associated with these features across the 1000 bootstrap samples. A comparison of the outputs of models M1 and M1ʹ is shown in Figure 5.

(18) $$\begin{array}{cc} D_{M1^{\prime }} & =\;3.243\;\left(\pm 1.251\right)\times \log_{10}\left(\mathrm{SUVmax}\right)\\ & \;+2.070\left(\pm 0.745\right)\times \log_{10}\\ & \;\left(\mathrm{HYPODENS}E_{20\;\mathrm{HU}}\cup \mathrm{INACTIV}E_{\mathrm{FDG}}\;V\right)\\ & \;+0.940\;\left(\pm 0.907\right)\times \mathrm{SHAP}E_{\mathrm{ELONGATION}}\\ & \;-0.468\;\left(\pm 0.482\right) \end{array}$$

## 4 DISCUSSION

In this study, we proposed a method to identify and characterize the tumor sub‐regions that drive the predictions of models built using engineered features. Indeed, even if engineered radiomic features are mathematically well defined, their interpretation often remains challenging. Their combination in a multivariate model further complicates this interpretation task. Our approach is based on engineered features calculated at the voxel level using a sliding window, followed by the averaging of voxel‐based feature values over the ROI to get one feature value per ROI for subsequent modeling. When using generalized linear models such as logistic regression, the backprojection of the model coefficients in the voxel space yields a decision map for each patient. These maps preserve the probabilistic information that is captured in the ROI space. The logistic activation of the mean voxel decision value within the ROI, added to the linear combination of the features that cannot be mapped, is equal to the modeled probability of belonging to class 1 for each patient. As such, the resulting decision maps are directly related to the models they are mapping, and partially show the marginal contribution of each and every voxel in the patient ROI to their modeled risk of metastasis occurrence. In the case of the combination of voxel‐level features with features with no local counterpart such as shape features, the decision maps only explain part of the model. Nevertheless, by exhibiting the most locally contributing voxels and analyzing this jointly with the input images, the proposed RDMs increase the interpretability of the models.

Technically, our approach is comparable to the class activation map (CAM) associated with deep learning models.^7^ Indeed, the principle of CAM is to use the global average pooling in a convolutional neural network architecture to compute the average of all voxels in the last feature maps in order to produce the output based on a unique fully connected layer. Once the network is trained, the backprojection of the linear coefficients of this layer yields the CAMs. In case of binary classifications, a fully connected layer with a sigmoid activation corresponds to a logistic regression, making our method close to the deep learning CAM approach. The use of the average feature value across voxels in the (last) feature maps enables training the models on images or ROI of different size, and also makes the classification models invariant by translation. This is a key difference compared to other saliency approaches that do not use global average pooling and connect one or several layers to all the voxels of the last feature maps. Besides, when using several classification layers, the use of the backpropagated gradients is not without risk when interpreting them as the importance of the voxels.^13^, ^14^

Our approach uses a sliding window of chosen dimensions to compute the local features for each and every voxel in the input images to obtain decision maps of relatively fine resolution, directly comparable to the input images for joint analysis and allowing the identification of sub‐regions within the tumor ROI. The identification of sub‐regions might be useful in the context of dose painting in radiation therapy for instance. This contrasts with most of the fully downsampling strategies used in deep learning that yield sparse or coarse saliency maps. In terms of interpretability, selecting the receptive field of the model by setting the size of the sliding window defines how far the model captures information around each voxel.

Compared to the classical ROI‐based extraction, the impact of random noise and artefacts or bias on voxel‐wise radiomics has been studied in Bernatowicz et al.^41^ The authors concluded that voxel‐level feature extraction is more affected than ROI‐level feature calculation. Indeed, when extracting a global radiomic feature for a given patient, the voxels within the ROI are aggregated to yield the scalar value of the feature. As the sliding window of our approach is smaller than the ROI, the aggregation involves less voxels and is thus more affected by noise and artifacts. A fair comparison between the two approaches would need to first average (aggregate) the voxel‐wise feature values over the ROI (as we do for our modeling step) before being compared with the ROI‐level feature values. Although this remains to be demonstrated, it is expected that such averaging will smooth out the impact of random noise and artifacts. Yet, when backprojecting the decision functions at the voxel level after modeling, we go back to a space that is more prone to artifacts and noise. However, this can be an asset: by highlighting patterns possibly due to noise or bias, the approach will make them detectable, avoiding misleading interpretation, while such bias might remain undetected in a complete ROI‐based approach.

Our method also presents similarities with multiple instance learning approaches,^42^ in which each classified individual is represented by a “bag of instances.” Here, the bag corresponds to the patient's ROI, where the voxels inside this ROI represents the multiple instances.

RDMs use engineered features. Despite this can be seen as a lack of optimization compared to deep learning approaches, this makes our method more suitable for small cohorts. More importantly, deep features, although optimized for a problem and defined locally, do not have an explicit mathematical definition.^12^ The associated mapping methods thus make it possible to locate the relevant information but they do not explain how the signal is captured. Thanks to the engineered nature of the features from which they are made up, RDMs are mathematically well defined for each voxel inside the ROI, facilitating their quantitative interpretation.

Our method also handles models relying on features without any local meaning, such as shape features. This is thus compatible with models involving even non‐imaging features, such as clinical or genomic features. Such holistic models could still benefit from our proposed RDMs that characterize the local image patterns partially contributing to the decision.

More generally, we can see radiomic models as tools to automate and help physicians in patient management. Deploying such models in practice requires high generalizability, that is ability of being applicable in a multi‐center context^43^ and built with a sufficient amount of data representative of the population. Beyond the objective of deploying a predictive model in practice, we can use models to generate intuitions and new insights through their semantic interpretation. This would make it possible to benefit from the information present in the images even with small and heterogeneous datasets incompatible with the deployment of predictive models, and enhance our understanding of the relationship between the image content and what we want to predict. This was the main goal of the present study, as opposed to building the most accurate model given all information available, in which case we would have included all four imaging modalities in our model. Building trustworthy models implies to consider different aspects of their transparency.^44^ The “algorithm transparency” corresponds to how the algorithm learns a model from the data. “Global, holistic model interpretability” aims at understanding how the model make predictions (e.g., which features are important, what links do they have together and with the target, how can we explain the link between the decision of the model and its inputs). Finally, “local model interpretability” corresponds to examining the prediction of the model at the individual scale (e.g., what is the output for a given patient and why). These three aspects are covered by our method based on an intrinsically fully interpretable logistic regression model.

In terms of prediction performance, the PET/CT model M1 yielded higher ROC AUC and lower Brier score loss than the MRI model M2. Nonetheless, M2 yielded higher ROC AUC than SUVmax, which was the “conventional” biomarker with the highest ranking performance. The performance of M2 together with the necrotic sub‐regions highlighted by the associated RDMs underline the importance of necrosis assessment to evaluate the risk of metastasis occurrence in STS. The necrotic sub‐regions were identically observed in the RDMs of the PET/CT model M1, which also displayed high decision values in sub‐regions exhibiting high FDG uptake. This suggests that the combination of necrosis and highly metabolically active tumor regions at baseline is highly predictive of the risk of metastasis occurrence. This interpretation was further supported by the design of the simpler surrogate model M1ʹ, in which SUVmax and the hypodense or non‐metabolically active tumor volume were automatically selected to produce results close to those obtained with M1 (with a common shape feature measuring the elongation of the tumor). Due to the small size of the dataset, OOB ROC AUC distributions have large confidence intervals. The differences between the models and the biomarkers thus have low statistical power and significance (bootstrap *p*‐value >0.05).

Our findings are consistent with image‐based studies,^45^, ^46^, ^47^ as well as with the STS grading systems based on the biopsy and showing ability to predict metastasis development and mortality. Indeed, the National Cancer Institute STS grading system relies on histology, location, and tumor necrosis. The French Fédération National des Centres de Lutte Contre le Cancer grading system is also based on tumor differentiation, mitotic activity, and tumor necrosis.^48^ In addition to the necrosis that can be assessed in FDG PET/CT by identifying hypometabolic and hypodense signal, Rakheja et al.^49^ related FDG uptake to histological features and mitotic activity and showed a significant positive correlation between mitotic count and SUVmax.

Our results are also consistent with the interpretations given by Vallières et al.^21^ when building models from the same STS dataset. From their univariate correlation results and their multivariate models, they suggested that the presence of a necrotic sub‐region inside the tumor ROI would be associated with a higher risk of metastasis. They also suggested that the presence of sub‐regions with high FDG uptake may play an important role in the characterization of high‐risk tumors. These interpretations were based on the mathematical definition of the engineered features. However, the fused images from which the features were extracted did not allow us to identify precisely what part of the information was captured by each modality. In addition, the biological interpretation of their results was not supported by any local importance map and was thus limited to a global interpretation.

Our results are thus consistent with up‐to‐date knowledge of STS and the proposed method did not yield any new discovery of predictive image patterns in this medical context. Yet, this consistency suggests that this completely data‐driven method could be used when little is known about the tumor features associated with an outcome to highlight sub‐regional patterns that drive the model decision, which may facilitate the emergence of new biological or medical hypotheses.

This study has some limitations. Some of these are related to the modeling pipeline. First, despite their great efficiency to find a good subset of features, sequential feature selection approaches are prone to overfitting due to their intrinsic multiple‐comparison mode of operation. Moreover, although it has been shown that this is most of the time not critical when using simple models,^50^ the evaluation of the performance can lead to an optimistic bias when carried out simultaneously with hyperparameter optimization without performing the so‐called nested cross‐validation. Unfortunately, the number of available patients is often not sufficient in radiomic studies to perform a nested approach, as in the cohort of 51 patients analyzed here. As our goal was to demonstrate how to get informative importance maps rather than to deploy a predictive model, we used a permutation test to ensure the patterns that were captured by the models were not noise.

Another limitation is that the average‐aggregated feature values over the ROI are not necessarily equal to or even correlated with the feature values directly calculated from the ROI. This makes our mapping approach incompatible with already published radiomic signatures, that are almost always calculated directly from the ROI. In addition, some engineered radiomic features remain challenging to interpret despite their precise mathematical definition and this complexity is only compensated here by the local identification of the relevant information without any loss in spatial resolution compared to the original images. It might still be useful to develop a methodology to easily convert a complicated radiomic signature into a simpler and more robust one that even might generalize better. A potential limitation is also that using a sliding window could miss some global features in the ROI, for instance features that measure some joint information between voxels that are at a distance higher than the maximum distance in the sliding window. Yet, we empirically observed that most of these features had values highly correlated with the tumor volume or shape features (results not shown). Therefore, by including the tumor volume and shape features in our model, we do not expect to miss substantial information.

The identification of tumor sub‐regions associated with any classification problem might enable a better understanding of the spatial components of the pathology for each patient. If the identification and the interpretation of these sub‐regions can be associated with causal relationships, one might be able to locally adapt and personalize the treatment of each patient given the phenotypic expression of his or her disease, as Reuzé et al.^51^ proposed in the context of radiotherapy.

## 5 CONCLUSION

We have described a generic method based on locally calculated engineered radiomic features to spatially and quantitatively characterize the sub‐regions and biological signal driving the prediction of a radiomic model. When the number of data is limited, we demonstrate how that method yields a consistent spatial and quantitative interpretation of radiomic models and identifies potential biomarkers useful for patient classification or stratification. As being technically applicable to any problem dealt with using engineered radiomics, this method could help to increase our understanding of the relevant information brought by medical images when little is known about the tumor image‐based features associated with the question of interest. In addition, in the future, it could assist in the identification of sub‐regions strongly associated with poor outcome that should be targeted for improved patient management.

## CONFLICTS OF INTEREST

Irène Buvat's lab receives overhead fundings from DOSIsoft SA as part of Thibault Escobar PhD project. Thibault Escobar is employed by DOSIsoft SA as a PhD student with the Laboratoire d'Imagerie Translationnelle en Oncologie (LITO), Institut Curie. Sébastien Vauclin and Pascal Pineau are the DOSIsoft SA advisors of Thibault Escobar. All authors have no other relevant links of interest to disclose.

## Supporting information



Supporting Appendix

## Data Availability

The data that support this study are available from the corresponding author upon request.
