# Supplementary material for: Voxel‐wise supervised analysis of tumors with multimodal engineered features to highlight interpretable biological patterns
Source: Med Phys. 2022 Apr 21;49(6):3816–29. doi: 10.1002/mp.15603 (PMC9325536; doi:10.1002/mp.15603)
Supplement: Supplementary file 1 — Supporting Appendix [file MP-49-3816-s001.pdf]

Voxel-Wise Supervised Analysis of Tumors with Multimodal Engineered Features to Highlight Interpretable Biological Patterns

Thibault Escobar[1,2], Sébastien Vauclin[2], Fanny Orlhac[1], Christophe Nioche[1], Pascal Pineau[2], Laurence Champion[1,3], Hervé Brisse[1,4], Irène Buvat[1]

SUPPLEMENTAL DATA

[1]Laboratoire d'Imagerie Translationnelle en Oncologie (LITO), Institut Curie, Inserm, Université Paris-Saclay, Orsay, France.
[2]DOSIsoft SA, Cachan, France.
[3]Department of Nuclear Medicine and Endocrine Oncology, Institut Curie, Saint-Cloud, France.
[4]Department of Medical Imaging, Institut Curie, Paris, France.

Correspondence
Thibault Escobar, Laboratoire d'Imagerie Translationnelle en Oncologie (LITO), Université Paris-Saclay, U1288 Inserm, Institut Curie, Bâtiment 101B, Rue de la chaufferie, 91405 Orsay, Île-de-France, France.
Email: thibescobar@gmail.com

EQUATION (S1). Detailed development of Equation (5).

$$
\begin{aligned}
\overline{DV^{(i)}} &= \frac{1}{Nv^{(i)}} \sum_{v=1}^{Nv^{(i)}} DV\left(X^{(i,v)}\right) \\
\overline{DV^{(i)}} &= \frac{1}{Nv^{(i)}} \sum_{v=1}^{Nv^{(i)}} \left(\beta^T X^{(i,v)} + \beta_0\right) \\
\overline{DV^{(i)}} &= \frac{1}{Nv^{(i)}} \sum_{v=1}^{Nv^{(i)}} \left(\beta^T X^{(i,v)}\right) + \frac{1}{Nv^{(i)}} \sum_{v=1}^{Nv^{(i)}} \beta_0 \\
\overline{DV^{(i)}} &= \frac{1}{Nv^{(i)}} \sum_{v=1}^{Nv^{(i)}} \left(\beta^T X^{(i,v)}\right) + \beta_0 \\
\overline{DV^{(i)}} &= \frac{1}{Nv^{(i)}} \sum_{v=1}^{Nv^{(i)}} (\sum_{p=1}^{Np} (\beta_p x_p^{(i,v)})) + \beta_0 \\
\overline{DV^{(i)}} &= \frac{1}{Nv^{(i)}} \sum_{p=1}^{Np} (\sum_{v=1}^{Nv^{(i)}} (\beta_p x_p^{(i,v)})) + \beta_0 \\
\overline{DV^{(i)}} &= \frac{1}{Nv^{(i)}} (\sum_{p=1}^{Np} (\beta_p \sum_{v=1}^{Nv^{(i)}} x_p^{(i,v)})) + \beta_0 \\
\overline{DV^{(i)}} &= \sum_{p=1}^{Np} (\beta_p (\frac{1}{Nv^{(i)}} \sum_{v=1}^{Nv^{(i)}} x_p^{(i,v)})) + \beta_0 \\
\overline{DV^{(i)}} &= \sum_{p=1}^{Np} (\beta_p g_p^{(i)}) + \beta_0 \\
\overline{DV^{(i)}} &= \beta^T G^{(i)} + \beta_0 \\
\overline{DV^{(i)}} &= D\left(G^{(i)}\right) \\
\overline{DV} &= D \qquad (S1)
\end{aligned}
$$

TABLE S1. Used radiomic features.

**Type of feature (number of features)**	**Illustration**	**Features**
First order statistic (18)*	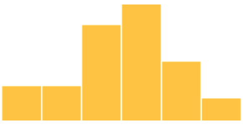	Energy, Total energy**, Entropy, Minimum, 10th percentile, 90th percentile, Maximum, Mean, Median, Interquartile range, Range, Mean absolute deviation (MAD), Robust mean absolute deviation (rMAD), Root mean squared (RMS), Skewness, Kurtosis, Variance, Uniformity
Gray level cooccurrence matrix (GLCM) (24)*	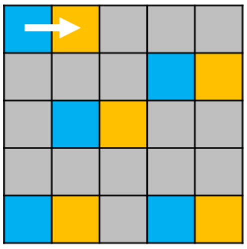	Autocorrelation, Joint average, Cluster prominence, Cluster shade, Cluster tendency, Contrast, Correlation, Difference average, Difference entropy, Difference variance, Joint energy, Joint entropy, Maximum probability, Maximum correlation coefficient (MCC), Informational measure of correlation (IMC) 1, Informational measure of correlation (IMC) 2, Inverse difference moment (IDM), Inverse difference moment normalized (IDMN), Inverse difference (ID), Inverse difference normalized (IDN), Inverse variance, Maximum probability, Sum entropy, Sum of squares
Gray level run length matrix (GLRLM) (16)*	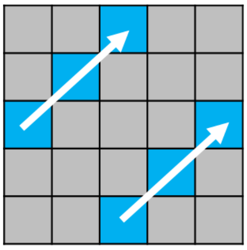	Short Run Emphasis (SRE), Long Run Emphasis (LRE), Gray Level Non-Uniformity (GLNU), Gray Level Non-Uniformity Normalized (GLNUN), Run Length Non-Uniformity (RLNU), Run Length Non-Uniformity Normalized (RLNUN), Run Percentage (RP), Gray Level Variance (GLV), Run Variance (RV), Run Entropy (RE), Low Gray Level Run Emphasis (LGLRE), High Gray Level Run Emphasis (HGLRE), Short Run Low Gray Level Emphasis (SRLGLE), Short Run High Gray Level Emphasis (SRHGLE), Long Run Low Gray Level Emphasis (LRLGLE), Long Run High Gray Level Emphasis (LRHGLE)
Gray level dependence matrix (GLDM) (14)*	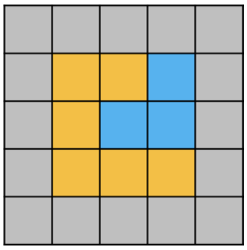	Small Dependence Emphasis (SDE), Large Dependence Emphasis (LDE), Gray Level Non-Uniformity (GLNU), Dependence Non-Uniformity (DNU), Dependence Non-Uniformity Normalized (DNUN), Gray Level Variance (GLV), Dependence Variance (DV), Dependence Entropy (DE), Low Gray Level Emphasis (LGLE), High Gray Level Emphasis (HGLE), Small Dependence Low Gray Level Emphasis (SDLGLE), Small Dependence High Gray Level Emphasis (SDHGLE), Large Dependence Low Gray Level Emphasis (LDLGLE), Large Dependence High Gray Level Emphasis (LDHGLE)
Neighbouring gray tone difference matrix (NGTDM) (5)*	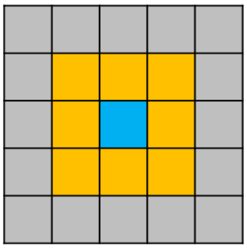	Coarseness, Contrast, Busyness, Complexity, Strength
Shape (14)	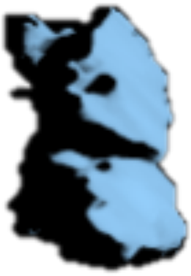	Mesh volume, Voxel volume, Surface area, Surface area to volume ratio, Sphericity, Maximum 3D diameter, Maximum 2D diameter (slice), Maximum 2D diameter (column), Maximum 2D diameter (row), Major axis length, Minor axis length, Least axis length, Elongation, Flatness

Mathematical definitions can be found at *https://pyradiomics.readthedocs.io/*

*Except for shape, features were extracted at the voxel level using a 3D sliding kernel of chosen dimensions as illustrated below. In this illustrative example, a feature map is computed using a 3 × 3 × 3 voxels kernel and the result is assigned to the central voxel of this window in the resulting 3D feature map. This process is repeated for all features and all voxels inside the ROI.

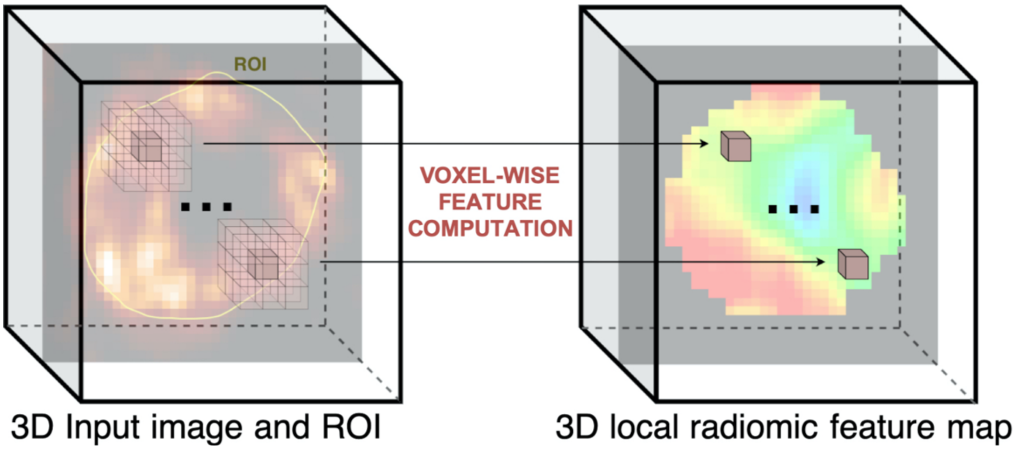

**Since the features are calculated from a kernel of constant size, FIRST-ORDER-TOTAL-ENERGY is equivalent to FIRST-ORDER-ENERGY.

TABLE S2. VIF of the PET/CT and MRI multicollinearity-based selected features (darker background corresponds to the features that were finally selected in the models).

**Modality**	**Feature**	**VIF**
PET/CT	PET-FIRST-ORDER-SKEWNESS	9.45
	CT-GLCM-CLUSTER-PROMINENCE	8.01
	PET-GLCM-INVERSE-VARIANCE	7.09
	CT-FIRST-ORDER-SKEWNESS	6.87
	PET-GLRLM-LRLGLE	6.85
	CT-GLCM-CORRELATION	6.60
	PET-GLCM-CORRELATION	6.60
	SHAPE-SHPERICITY	6.55
	CT-FIRST-ORDER-90-PERCENTILE	6.35
	PET-NGTDM-STRENGTH	6.33
	PET-NGTDM-COARSENESS	6.03
	CT-FIRST-ORDER-ENERGY	5.80
	PET-GLDM-SDLGLE	5.39
	PET-FIRST-ORDER-MINIMUM	5.16
	SHAPE-MESH-VOLUME	4.93
	SHAPE-MESH-FLATNESS	4.84
	SHAPE-MESH-ELONGATION	3.60
	PET-GLCM-CLUSTER-SHADE	3.48
	PET-GLCM-IDMN	3.38
	CT-GLCM-IDN	3.26
	CT-GLDM-LDLGLE	3.05
	CT-GLDM-SDHGLE	2.94
	CT-GLDM-SDLGLE	2.92
	CT-GLDM-LDHGLE	2.59
	CT-NGTDM-COARSENESS	2.15
MRI	fat-supressed-T2-GLCM-MCC	8.87
	SHAPE-FLATNESS	7.56
	fat-supressed-T2-FIRST-ORDER-SKEWNESS	7.29
	SHAPE-SURFACE-AREA	6.75
	fat-supressed-T2-FIRST-ORDER-ENERGY	6.29
	fat-supressed-T2-GLDM-SDHGLE	6.07
	fat-supressed-T2-GLDM-LDHGLE	5.78
	SHAPE-ELONGATION	5.78
	fat-supressed-T2-NGTDM-COARSENESS	5.48
	fat-supressed-T2-FIRST-ORDER-MEAN	5.44
	fat-supressed-T2-FIRST-ORDER-KURTOSIS	5.04
	T1-GLCM-INVERSE-VARIANCE	4.98
	T1-GLDM-LGLE	4.41
	T1-GLDM-SDLGLE	3.96
	fat-supressed-T2-GLCM-CLUSTER-PROMINENCE	3.73
	fat-supressed-T2-FIRST-ORDER-RMS	3.66
	fat-supressed-T2-GLDM-LGLE	3.65
	SHAPE-SPHERICITY	3.47
	T1-FIRST-ORDER-ENERGY	3.30
	T1-GLDM-DNU	3.27
	T1-GLDM-LDHGLE	3.25
	T1-FIRST-ORDER-SKEWNESS	3.17
	T1-GLCM-IMC1	2.78
	T1-NGTDM-BUSYNESS	2.76
	T1-NGTDM-COARSENESS	2.16
	T1-GLCM-CLUSTER-SHADE	2.13

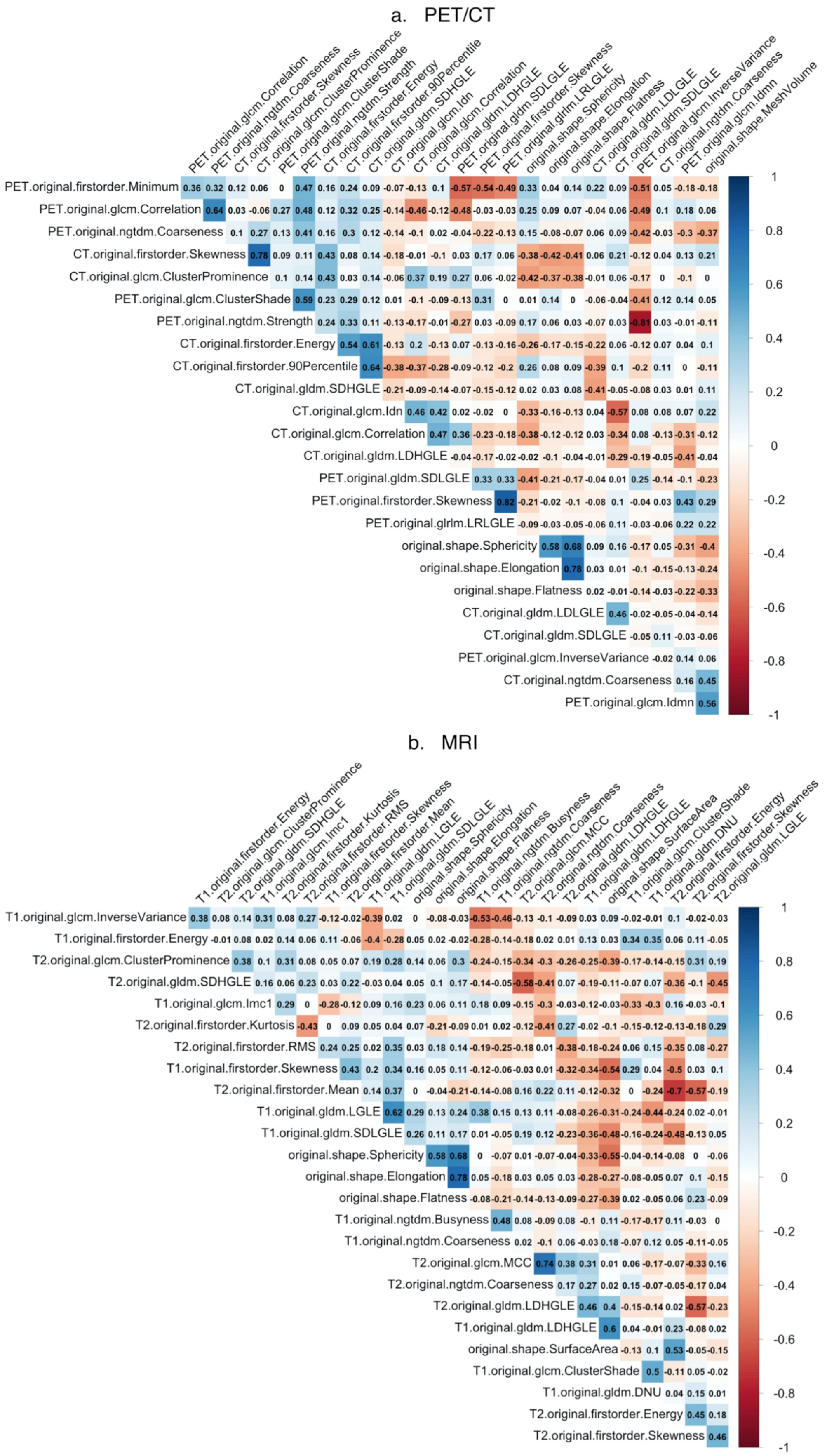

FIGURE S1. Pearson correlation matrices for PET/CT (a) and MRI (b) VIF-based selected features.

TABLE S3. Surrogate model features.

Feature	Definition	Modality
Anatomical tumor volume (ATV)	$Nv \times x \times y \times z$ *	CT
SUVmax	$\max(PET_{ROI})$	PET
Metabolic tumor volume (MTV)	$Nv_{>40\,\%\,SUVmax} \times x \times y \times z$	PET
Total lesion glycolysis (TLG)	$MTV \times \frac{1}{Nv_{>40\,\%\,SUVmax}} \times \sum_{Nv_{>40\,\%\,SUVmax}} PET_{ROI_{>40\,\%\,SUVmax}}$	PET
Non-metabolic volume (INACTIVE-FDG V)	$Nv_{<40\,\%\,SUVmax} \times x \times y \times z$	PET
Non-metabolic relative volume (INACTIVE-FDG rV)	$\frac{INACTIVE_{FDG}\,V}{ATV}$	PET/CT
Hypodense volume < 20 HU (HYPODENSE-20 HU V)	$Nv_{<20\,HU} \times x \times y \times z$	CT
Hypodense relative volume < 20 HU (HYPODENSE-20 HU rV)	$\frac{HYPODENSE_{20\,HU}\,V}{ATV}$	CT
Hypodense volume < 30 HU (HYPODENSE-30 HU V)	$Nv_{<30\,HU} \times x \times y \times z$	CT
Hypodense relative volume < 30 HU (HYPODENSE-30 HU rV)	$\frac{HYPODENSE_{30\,HU}\,V}{ATV}$	CT
Non-metabolic or hypodense < 20 HU volume (INACTIVE-FDG ∪ HYPODENSE-20 HU V)	$Nv_{<40\,\%\,SUVmax\ or\ <20\,HU} \times x \times y \times z$	PET/CT
Non-metabolic or hypodense < 20 HU relative volume (INACTIVE-FDG ∪ HYPODENSE-20 HU rV)	$\frac{INACTIVE_{FDG} \cup HYPODENSE_{20\,HU}\,V}{ATV}$	PET/CT
Non-metabolic or hypodense < 30 HU volume (INACTIVE-FDG ∪ HYPODENSE-30 HU V)	$Nv_{<40\,\%\,SUVmax\ or\ <30\,HU} \times x \times y \times z$	PET/CT
Non-metabolic or hypodense < 30 HU relative volume (INACTIVE-FDG ∪ HYPODENSE-30 HU rV)	$\frac{INACTIVE_{FDG} \cup HYPODENSE_{30\,HU}\,V}{ATV}$	PET/CT
Non-metabolic and hypodense < 20 HU volume (INACTIVE-FDG ∩ HYPODENSE-20 HU V)	$Nv_{<40\,\%\,SUVmax\ and\ <20\,HU} \times x \times y \times z$	PET/CT
Non-metabolic and hypodense < 20 HU relative volume (INACTIVE-FDG ∩ HYPODENSE-20 HU rV)	$\frac{INACTIVE_{FDG} \cap HYPODENSE_{20\,HU}\,V}{ATV}$	PET/CT
Non-metabolic and hypodense < 30 HU volume (INACTIVE-FDG ∩ HYPODENSE-30 HU V)	$Nv_{<40\,\%\,SUVmax\ and\ <30\,HU} \times x \times y \times z$	PET/CT
Non-metabolic and hypodense < 30 HU relative volume (INACTIVE-FDG ∩ HYPODENSE-30 HU rV)	$\frac{INACTIVE_{FDG} \cap HYPODENSE_{30\,HU}\,V}{ATV}$	PET/CT

*To allow for the computation of PET/CT-based features, we resampled all PET images on a common grid with their corresponding CT using nearest neighbor interpolation. Thus, x, y, and z are common to both modalities and represent the dimensions of the CT image. $Nv_{<condition>}$ is the number of voxels in the ROI that meet the condition noted as index.
